# Dentition and Its Derangements

**Published:** 1861-12

**Authors:** A. Jacobi


Dentition and its Derangements.—By *A. Jacobi*, M.D. —*Lecture VI.—Part 1.*—No part of the infantile organism is more exposed to injurious influences than the mucous membrane of the mouth, nor is there any which is more frequently observed to suffer. Traumatic injuries are not frequent, except those sometimes produced by sharp margins of teeth irregularly shaped; the more frequent affections are those resulting either from chemical influences, or from an excessive degree of temperature. The mucous membrane of the mouth is very irritable, being accustomed only to amniotic liquor in fœtal life, and to milk in the early stage of extra-uterine existence. Every change in the diet, therefore, the bad quality of the maternal or artificial nipples, the use of candy, sucking bags, or alcoholic beverages, coffee, or stimulants of whatever kind, will act as irritants, producing hyperæmia or inflammation in a more or less severe form. It is by no means common to observe very severe forms of stomatitis after all such preceding causes; on the contrary, the large majority of cases, including those depending on primary acute catarrh of the stomach, and the raising of a large quantity of gastric acid, so frequent in infantile age, are very mild. Nor are some of the most severe forms of stomatitis in adults often found in early age. Thus it is a peculiar fact that the influence of the external and internal use of mercury has little influence on the mucous membrane of the mouth, or the salivary glands, in infantile life. Whatever the consequences of the administration of mercurial preparations may be, salivation, or even a mild form of erythematous stomatitis, is seldom observed; in a large number of adult patients there will perhaps none be found who will not suffer from a certain amount of mercury, but of infants and children of even more advanced age, those who show mercurial symptoms are exceptions to the rule.

There are a number of indirect influences also observed to produce the common, or erythematous form of stomatitis. It will often be seen in dependence on, or in connection with traumatic injuries of the face, erysipelas, and hyperæmia and inflammation of the pharynx. It is further seen under the influence of many dyscrastic processes, as it is a very common symptom attending scarlatina, variola, morbilli, syphilis, and typhoid fever. It is frequently, as its causes will often continue or return, or be replaced by others, of long duration and obstinacy, like the pharyngeal hyperæmia and swelling in adults, and very generally proves a serious difficulty, although unattended by severe fever or deep seated anatomical disorganization of any particular organ. Injection, swelling, high temperature, and slightly reddened color of the mucous membrane, copious or suppressed secretion, and pain on being touched, are the usual symptoms of the common form of erythematous stomatitis.

A more severe form is that known by the name of aphthous stomatitis. The superficial layers of the epithelium are not thrown off during the hyperæmic swelling of the mucous membrane, as in erythematous stomatitis, but a real and visible change takes place in the anatomical structure of the follicles. There is a circumscribed, punctated, vascular injection around a follicle, which is gradually infiltrated by exudation. The consecutive swelling increases in proportion, the follicles will burst and exhibit a superficial erosion, or ulceration, and the adjacent mucous membrane will be sympathetically affected. Some of these cases, which are by no means very frequent, look very much like the vesicles of labial herpes, with the only exception that they are less accommodated on a certain small locality; some may be explained by mechanical injuries, some can not be explained at all. If it was not for those cases occurring in the first two months of life, so well described by Beduar, aphthous inflammation of the mouth would be a very rare disease; at all events the first stage will seldom come under observation, and usually the second stage, in which the vesicles are fully developed, is brought to your notice.

That dentition, that is, the protrusion of teeth through the gums, can have nothing to do with this form of stomatitis, is manifest from the fact that it occurs mostly in the earliest period where teeth protrude in but very rare and exceptional cases; and that, whenever it is seen in advanced age, no connection, either casual or as to time, can be found between the two. Much less can be said of all the forms of inflammation of the tongue, known to be the consequences of caustic substances, combustion, or the poisonous stings of insects; this parenchymatous glossitis has not even been supposed by the most ardent advocates of the universal danger of dentition to be the result of its influence. Nor are the most severe forms of disease of the mouth attributed to dentition, like noma, or scurvy, or diphtheritic inflammation. They are, like the usual forms of stomacace, in which fibrinous exudations are deposed into the superficial layers of the mucous membrane, with an immediate tendency to gangrenous decomposition, well known to be not only the result of a local affection, but more so of a general decomposition of the blood. They are to be considered as the local symptoms of a general disease, the former being entirely subordinate; to say nothing of the age in which they occur by preference. Diphtheritic inflammation will occur in any age, but mostly between the first and third, at all events rarely before and during the protrusion of the first incisors; scurvy, noma, and stomacace are mostly seen in a somewhat advanced age, between the fourth and tenth years of life. In these, the local affection is something; but the larger amount of the symptoms and of danger depends on the general character of the disease.

There is another form of disease, on which nearly the same remarks may be made. Inflammation of the parotid gland, both idiopathic and symptomatic, is not a very uncommon disease, except in the age of dentition. Idiopathic parotitis will usually occur as an epidemic disease, in a similar manner as diseases of the larynx, or pneumonia, will appear as an epidemic, from some causes not perfectly understood, but depending on season and the constitution of the atmosphere; this idiopathic form is seldom seen both in the first year of life and in sensile age. The symptomatic form, which will usually terminate in suppuration, and is observed in certain epidemics of typhoid fever, cholera, septicohæmia, and in some of variola, measles, dysentery, and pneumonia, is very rarely observed in small children; and therefore, among the cases of the above-named diseases, dentition is out of the question, with the exception, perhaps, of an occasional slight swelling of the parotid gland, brought on by the contiguity of the mucous membrane. I certainly do not deny the possibility of erythematous stomatitis occurring during the protrusion through abnormal gums, of perhaps an abnormal tooth, in an abnormal direction, and in an abnormally irritable child—one or more of these conditions being together, and therefore admit that a mild parotitis may sometimes occur in a casual connection with dentition ; but what I deny, and have attempted to prove by the illustration of the physiological process of dentition, is this, that diseases depending on this process are not the rules, but the exceptions. At all events, not even the slightest erythematous stomatitis must be permitted to go on the plea of dentition, unless there is a local hyperæmia of the gums, the seat of the supposed cause of disease, corresponding with the more general affection. I lay the more stress on this, as I believe I have shown by the numerous causes of stomatitis exhibited to you, that we need not be at a loss to find a cause in any given case, if we are competent to form a differential diagnosis. As long as there is certainty, we had better not resort to hypothesis or conjecture.

If a large number of cases of stomatitis was the result of dentition, why is it that a uniform mode of treatment, if any is resorted to, has been accepted in these cases, relating to, and dependent on this cause? And why is it, that if any uniform treatment has been accepted, and is recommendable, it is just such as has no connection whatever with dentition? And why further is it, that having no regard whatever to either teeth or gums, it is so uniformly successful? I speak of the chlorate of both potassa and soda, the effect of which in all these cases can no longer be doubted. It has long been a matter of difficulty after it had been largely introduced into practice, since the times of Hunt, West, Isambert and others, to decide whether the effect was local or general. But the experiments of Gamberini and Semmola show, that the local effect of chlorate of potassa in mercurial stomatitis is very little, if any ; but that the same remedy administered in sugar-coated pills, has a satisfactory effect. My own experience has led me to the same conviction, although, if any local effect is produced, it could be done by the chlorate being transmitted into and secreted by the saliva.

Another of those diseases often enumerated among the consequences of dentition is that sometimes called membranous stomatitis, now better known by the French name of "muguet."

Muguet is an affection which a few facts will prove to have not the slightest connection with dentition. It has been generally observed in new-born infants, or in those but a few weeks old, but it is occasionally met with in more advanced life, even in adults suffering from exhausting and fatal diseases, towards the close of life. It is known by the occurrence of whitish or greyish, cream or cheese-like deposits of variable sizes, on the mucous membrane of the upper part of the digestive tract; they will be found on the lips, tongue, cheeks, pharynx, even in the larynx and œsophagus, but never in the stomach. One of its prominent symptoms, as described by adults, is a burning pain in the mouth, corresponding with the local affection; that infants suffer in a similar manner, is proved by their crying on being touched, and by their unwillingness to take the breast or swallow. Where no deposit happens to be seen, the mucous membrane appears injected, dry and smooth, and but little mucus and saliva is secreted. In perhaps every case diarrhea has been observed; so regularly indeed, that Vallaix speaks of diarrhea as one of the common and almost pathognomonic symptoms of muguet. It is, however, probable that its cause is to be sought for in the impaired digestion, want of mastication, absence of saliva, and affection of the mucous membrane generally.

The enumeration of a number of symptoms does not explain the nature of a morbid process, or a pathological deposit; and nothing but a description of the pultaceous deposits on the mucous membrane will illustrate the morbid change taking place in the mouth. They consist of the mucus of the lining membranes, of old and new epithelial cells, of fat globules, particles of food more or less decomposed, and finally, of microscopical fungous growths of different size, with sharp outlines and indentations, from which equally composed thalli will originate, to such a number sometimes as to form a network of dendritic parasitic tissue. The fungus was discovered by Robin, and called *oïdium albicans*, and has been described by Laycock, Gubler, and a host of other medical writers. It is not known in any form differing from that found in the mouth, and it is probable that it is, as such, contained in the air, and deposited at the entrance of the digestive organs; at least no other opportunity for its occurrence on the mucous membrane of the mouth is possible. It may be transmitted by the atmosphere, or transplanted from one individual to another by direct contact, by the use of the same spoon, etc.

But it will not always develop itself with the same readiness, certain conditions being required. They depend on an acid condition of the mucous secretion of the mouth, a certain dryness and injection of the mucous membrane, feebleness of mastication, and easy access of air. It is important to observe, that the secretion (as far as it is kept up) of the mucous membrane of the mouth is acid instead of alkaline. It is very frequently found in infants whose mouths are not kept so clean as they ought to be, who are accustomed to sleep while, or immediately after, taking the breast, and retaining milk in their mouth, which soon is decomposed and acid. Muguet is therefore often found in foundling hospitals, where the inmates receive but little care, and uncleanliness is almost the general rule. Where proper cleanliness is strictly enforced, no muguet will appear, because no parasitic fungus is allowed to settle and form a crust of pultaceous matter. Thus pure water is both the best prophylactic and curative agent; the only thing worth adding is a small quantity of alkaline substance, chlorate of potassa or soda, carbonate of potassa or soda, biborate of soda or chloride of sodium. The mouth of every infant ought to be washed out after each meal, to be certain that no deposit remains on the mucous membrane. Where such has been the case, the local treatment alluded is perfectly sufficient. The deposit is found in the superficial layers of the epithelium; it seldom reaches the deeper ones, and scarcely ever implicates the lining membrane itself. Thus cleanliness will remove the affection; the surface sometimes bleeds when the deposit is rubbed off. The addition of sugar, rose-honey, or syrup, to the water (or weak alkaline solution), must be strictly avoided; these substances will adhere to the lining membrane and themselves undergo decomposition and prove a source of new difficulties.

The occurrence of muguet, then, is a mere accident, and has no intrinsic connection either with a distinct morbid process, or with any certain period of early infantile development. It is no more characteristic of any constitutional disease, or general condition of the system, than tinea favosa on any part of the surface, which may be communicated from either man or animal, or scabies. You readily perceive that there is no shadow of a reason to search for any connecting link between the formation and protrusion of teeth and the accidental peculiar deposit on the mucous membrane of the mouth, called muguet, which years ago could be taken for a special kind of exudative stomatitis, but is now well understood.—*American Medical Times.*

